# BGDB: a database of bivalent genes

**DOI:** 10.1093/database/bat057

**Published:** 2013-07-26

**Authors:** Qingyan Li, Shuabin Lian, Zhiming Dai, Qian Xiang, Xianhua Dai

**Affiliations:** Department of Electronics, School of Information Science and Technology, Sun Yat-Sen University, Guangzhou 510006, China

## Abstract

Bivalent gene is a gene marked with both H3K4me3 and H3K27me3 epigenetic modification in the same area, and is proposed to play a pivotal role related to pluripotency in embryonic stem (ES) cells. Identification of these bivalent genes and understanding their functions are important for further research of lineage specification and embryo development. So far, lots of genome-wide histone modification data were generated in mouse and human ES cells. These valuable data make it possible to identify bivalent genes, but no comprehensive data repositories or analysis tools are available for bivalent genes currently. In this work, we develop BGDB, the database of bivalent genes. The database contains 6897 bivalent genes in human and mouse ES cells, which are manually collected from scientific literature. Each entry contains curated information, including genomic context, sequences, gene ontology and other relevant information. The web services of BGDB database were implemented with PHP + MySQL + JavaScript, and provide diverse query functions.

**Database URL:** http://dailab.sysu.edu.cn/bgdb/

## Introduction

Embryonic stem (ES) cells have the potential to differentiate into every tissue type of the body, and offer an important model for examining transitions of cellular identity in animals (1). It has been suggested that the potential is related to specific histone modifications or characteristic chromatin structure (2–4). Epigenetic regulation of gene expression is thought to be mediated partly by post-translational modifications of histones, which in turn establish different domains of active and inactive chromatin structures. The core histones have dozens of different modifications, including acetylation, methylation, phosphorylation and ubiquitylation. Histone H3 methylations of lysine 4 (K4) and lysine 27 (K27) have been shown to relate with active and repressed states, respectively (5). These methylations are catalyzed by Trithorax- and Polycomb-group proteins and play key roles in lineage-specific developmental functions (6). Trithorax-associated H3K4 trimethylation (H3K4me3) positively regulates transcription by recruiting nucleosome remodeling enzymes and histone acetylases (7–9), whereas Polycomb-associated H3K27 trimethylation (H3K27me3) negatively regulates transcription by promoting a compact chromatin structure (10, 11).The colocalization of these H3K4 and H3K27 histone methylations, termed ‘bivalent domains’, was found in ES cells by mapping mouse genome (12, 13). This modification pattern is observed in clusters of homeobox genes and other genes related to early embryonic development (12). The bivalent domains are proposed to silence key developmental genes in ES cells while keeping them poised for later activation, and these developmental genes marked by bivalent modifications are dubbed as bivalent genes (14). Whole-genome mapping found that H3K4me3 peaks were enriched in the region within 2 kb of the TSS of RefSeq annotations, and H3K27me3 peaks were also enriched in a band centered around the TSS with a greater width; moreover, most H3K27me3 peaks localized on promoters that were already marked with H3K4me3, suggesting that bivalent modifications on the same promoter is a rule in ES cells rather than an exception (15).

Genome-wide analyses of H3K4me3 and H3K27me3 in human ES cells and mouse ES cells identified several thousand genes marked with both trimethylation (15–20). These studies used diverse experimental approaches, such as hybridization, whole-genome microarrays (15), ChIP coupled with paired-end ditag sequencing (16) and single-molecule sequencing (18). Despite different ES cell lines and varied experimental methods used in these studies, they show remarkable consistency in genes marked with both H3K4me3 and H3K27me3. The high degree of consistency indicates that these data are reliable, especially for genes with bivalent domains identified by at least two independent experiments.

Since recent advances in high-throughput techniques such as genomic tiling microarrays and deep sequencing have discovered vast number of bivalent genes, it is an urgent topic to collect the experimental data and provide an up-to-date compressive resource for the community. Given these considerations, we have developed a novel database called ‘Bivalent Genes Database’ (BGDB) to store the sequence of bivalent genes and associated information from all studies published to date. In BGDB database, we manually curated 3913 bivalent genes in human ES cells and 2984 genes in mouse ES cells (Table 1), including the primary references and other annotations of these genes. Furthermore, we found 1604 genes have the same gene name in human and mouse ES cells (Table 1). Additionally, based on the gene ontology (GO) annotations, we analyzed the functional diversities and regulatory roles of bivalent genes. Taken together, the BGDB might be an integrated resource for bivalent genes and provide valuable information not only to stem cell biologists but also to researchers generally interested in gene expression regulation.

**Table 1.** Data statistics of the BGDB

Organism	Gene number	Percentiles (%)
*Homo sapiens*	3913	56.7
*Mus musculus*	2984	43.3
Total	6897	100
Both^a^	1604	23.3

^a^Genes with the same name in both *Homo sapiens* and *Mus musculus* ES cells.

## Database construction and content

**Figure 1.**
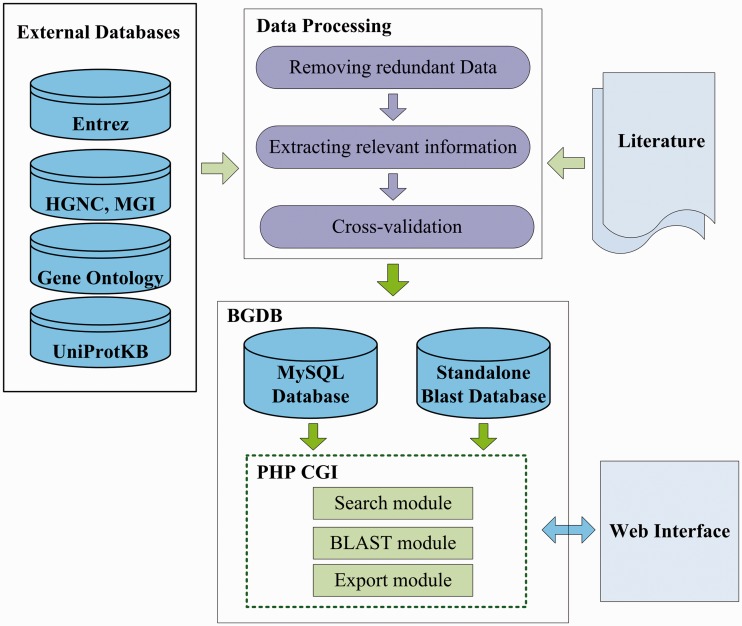
The data generation flow of the BGDB database.

The primary motivation of our BGDB is to collect and maintain a high quality bivalent genes database, which serves as an integrated, classified and well-annotated bivalent genes resource. The data generation flow of the BGDB is briefly illustrated in Figure 1. The generation flow is composed of three primary components: data processing, integration of external database and storing structural and functional annotation in database. To ensure the quality of BGDB database, we first performed a literature search of PubMed with major keywords ‘bivalent gene’ and ‘bivalent domain’. To avoid missing data, we next searched PubMed literature with keywords ‘H3K4 H3K27’ and ‘H3K4me3 H3K27me3’. Taking these four queries together, we collected and downloaded bivalent domain data for further manual review and curation. The search results are shown in Table 2.

**Table 2.** Search results in PubMed

Key words	Article number
Bivalent gene	820
Bivalent domain	405
H3K4 H3K27	142
H3K4me3 H3K27me3	204

The top 10 articles that contain most bivalent genes are shown in Supplementary Table S1

For curation of bivalent gene data from literature, we manually curated genes with bivalent domains and mapped the gene names to Entrez gene IDs. Then, we used Entrez gene IDs for BGDB to serve as the initial information to cross-link the same genes from different external databases. To avoid gene symbol ambiguity problems caused by synonyms of gene, we gained up-to-date official gene symbols from HGNC (21) and MGI (22) for human and mouse genes, separately. For better understanding the function and structure of these bivalent genes, we collected their extensive functional information as follows: basic gene information such as gene name, sequence and summary from Entrez gene database (23); gene product characteristics information from GO (24); and protein information related to gene from UnitProtKB (25).

**Figure 2.**
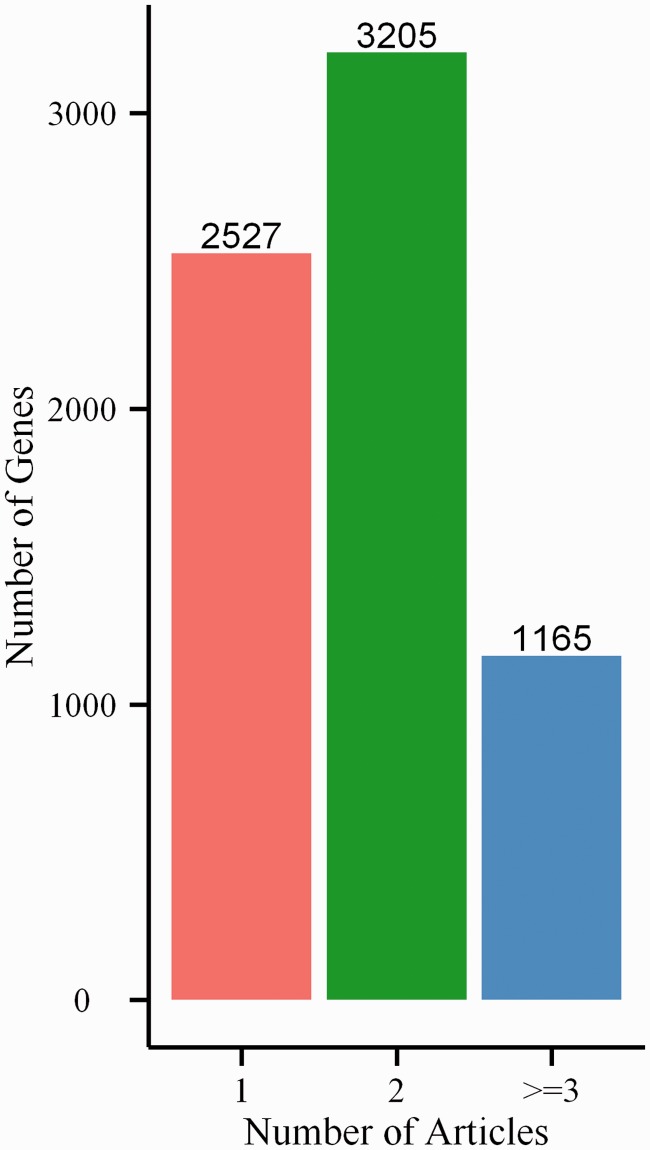
Number of bivalent genes found in 1, 2 and >2 references.

In BGDB database, we manually curated 6897 bivalent genes from scientific literature in PubMed. Not surprisingly, many bivalent genes were experimentally identified in at least two independent articles. There are 3205 (∼46.5%) genes that are cross-validated in two distinct studies and 1165 (∼16.9%) genes in more than two studies (Figure 2). Because ∼63% genes have passed cross-validation, this suggests the reliability of our database.

The annotations of each bivalent gene are described by the fields shown in Table 3. We build a MySQL relation database with two tables to store all the gene information. GO information, including GO ID, GO term and GO category, is stored in ‘Genes_go’ table. The ‘Genes’ table, which is defined as parent table, contains the other information. To enhance database normalization, we make the ‘Entrez ID’ field in ‘Genes_go’ as a foreign key and have it relate to the ‘Genes’ table. For providing a fast BLAST sequence alignment service, we also set up a local BLAST database and integrate the local BLAST application into web service. The web interface for searching and browsing was implemented by PHP and JavaScript.

**Table 3.** Description of fields used to annotate bivalent gene

Field name	Description
ID	Unique database identifier for the bivalent gene
Gene symbol	Approved symbol for the bivalent gene
Gene full name	Approved full gene symbol
Gene type	Biotype of the bivalent gene
Organism	Organism of the bivalent gene
Gene synonym	Other gene names used for the bivalent gene
Summary	Descriptive text about the gene
Reference	Articles that reported the bivalent gene
HGNC/MGI ID	HGNC ID for human bivalent gene, and MGI ID for mouse
Entrez ID	External link to Entrez gene
Ensembl ID	External link to Ensembl
UniProtKB ID	External link to UniProtKB
UCSC link	External link to UCSC
Gene ontology	The specific GO terms are listed by source of the information, category and term. Each GO term supports a link to the AmiGO browser
Genomic location	Genomic location of the bivalent gene
RefSeq ID	Reference sequence ID
Nucleotide sequence	Nucleotide sequence of the bivalent gene
Protein sequence	Protein sequence of the bivalent gene

## Usage

**Figure 3.**
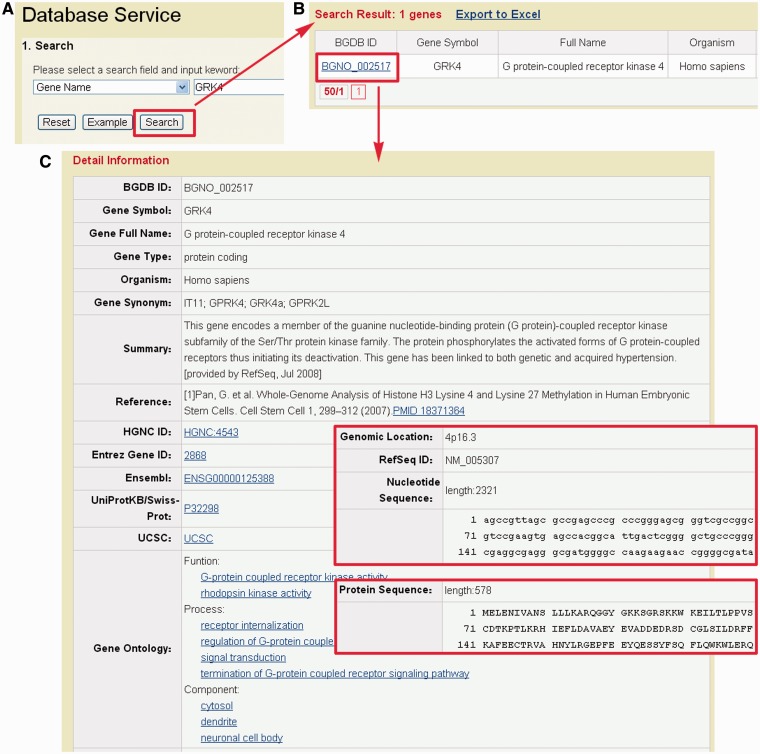
Representative screenshots of BGDB. (**A**) Users could input ‘GRK4’ for querying. (**B**) The results will be shown in a tabular format. Users could click on the BGDB ID (BGDB-002517) to view the detailed information. (**C**) The detailed information of bivalent gene GRK4. The nucleotide and protein sequence are also presented.

To facilitate the use of BDGB resource, we developed a user-friendly web interface for user to search and browse for content. The search page (http://dailab.sysu.edu.cn/bgdb/database.php) provides an interface for searching the BGDB database with several keywords such as gene symbol, gene alias, reference sequence ID or UniProt ID. For example, if a keyword ‘GRK4’ is inputted (Figure 3A), the query result will be shown in a tabular format, with the features of BGDB ID, gene symbol, gene full name, organism and gene alias (Figure 3B). By clicking the link of BGDB ID (BGNO_002517), the detailed information for gene GRK4 will be shown (Figure 3C). The gene information, including gene symbol, full name, summary and relevant references, is provided. The gene sequence, protein sequence, GO annotation, genomic location and some useful external links are also presented. All output columns are described in Table 3.

Furthermore, BGDB web interface provides three advanced options, including (i) batch search, (ii) BLAST search and (iii) browse function (Supplementary Figure S1). (i) Batch query: Using this function, users could query gene data for a batch of keywords at once with the results on one screen (Supplementary Figure S1A). (ii) BLAST search: Users can use an online BLAST interface to input an interested sequence in FASTA format and search against all nucleotide or protein sequences in our database (Supplementary Figure S1B). (iii) Browse: Instead of searching for specific genes, all entries of BGDB database could be listed by organism name (Supplementary Figure S1C).

For advanced bioinformatics users, all search results with related annotation, including nucleotide and protein sequence, GO and literature, are available to export with Excel format. Additionally, users could download the whole BGDB database with MySQL format (Supplementary Figure S1D).

## Discussion

Recent genome-wide analyses of H3K4me3 and H3K27me3 in human and mouse ES cells have revealed several thousands of bivalent genes, but mapping chromatin modifications across the genome is the first step toward understanding the mechanism of gene regulation in pluripotent stem cells. Because database development is important for further experimental and computational designs by providing a high-quality benchmark, we focus on data collection and manually curated 6897 bivalent genes in this work. With a large amount of bivalent gene information, we had the opportunity to analyze abundance and functional diversity of bivalent genes.

To gain insight into the functional distribution of GO, we conducted the enrichment tests on the bivalent genes in BGDB. Firstly the GO annotations in GAF 2.0 file format was downloaded from UniProt-GOA (24, 26), and secondly, the columns of gene symbol, GO ID, GO term and GO category were extracted and stored in the database. Then, taking account of the GO terms with genes directly associated to it, we mapped them to bivalent genes through gene symbol column that is provided in GO annotation. Using the human genome as background, we calculated overpresented biological processes, molecular functions and cellular components in bivalent genes of BGDB with the hypergeometric distribution (*P* < 0.001, calculated by Fisher's exact test). The five most enriched GO terms in each category are shown in Table 4. This analysis revealed several interesting results. For example, the four most overrepresented biological processes, such as anterior/posterior pattern specification, neuron differentiation, neuron migration and central nervous system development, indicate that bivalent genes are enriched for genes involved in system development and cell differentiation (Table 4), which is in accordance with the role of bivalent genes in ES cells. The enrichment result found here is consistent with the study reported previously (13). Also, four most abundant cellular components, such as axon, dendrite, neuronal cell body and postsynaptic membrane, suggest that bivalent genes are enriched in neuron compartments (Table 4). One possibility of this abundance is that neuron is an important cell type during ES cell differentiation. In addition, the statistical analysis of molecular functions shows that bivalent genes modulate enzyme activity and protein interaction ability (Table 4). For mouse bivalent genes in BGDB, we can draw a similar conclusion as above. The detailed information of top five most overrepresented GO terms of mouse bivalent genes is shown in Supplementary Table S2.

**Table 4.** The top five most enriched GO terms of biological processes, molecular functions and cellular components in human bivalent genes

Description of GO term	Bivalent gene	Genome	*E*-ratio^c^	*P*-value
	*n* (%)^a^^,^^b^	*n* (%)		
The top five most enriched biological processes				
Anterior/posterior pattern specification (GO:0009952)	70 (1.79)	102 (0.54)	3.31	2.72E-13
Neuron differentiation (GO:0030182)	46 (1.18)	81 (0.43)	2.74	3.30E-07
Negative regulation of canonical Wnt receptor signaling pathway (GO:0090090)	46 (1.18)	79 (0.42)	2.81	1.39E-07
Neuron migration (GO:0001764)	56 (1.43)	100 (0.53)	2.70	2.25E-08
Central nervous system development (GO:0007417)	58 (1.48)	108 (0.57)	2.59	3.57E-08
The top five most enriched molecular functions				
RNA polymerase II distal enhancer sequence-specific DNA binding transcription factor activity (GO:0003705)	62 (1.58)	108 (0.57)	2.77	2.00E-09
Metal ion binding (GO:0046872)	552 (14.11)	1050 (5.57)	2.53	8.35E-68
Sequence-specific DNA binding (GO:0043565)	260 (6.64)	536 (2.84)	2.34	2.70E-27
Transcription factor binding (GO:0008134)	116 (2.96)	280 (1.49)	2.00	2.18E-09
Protein dimerization activity (GO:0046983)	64 (1.64)	159 (0.84)	1.94	2.25E-05
The top five most enriched cellular components				
Voltage-gated potassium channel complex (GO:0008076)	48 (1.23)	69 (0.37)	3.35	1.26E-09
Axon (GO:0030424)	77 (1.97)	149 (0.79)	2.49	7.13E-10
Dendrite (GO:0030425)	82 (2.10)	178 (0.94)	2.22	1.18E-08
Neuronal cell body (GO:0043025)	104 (2.66)	223 (1.18)	2.25	1.05E-10
Postsynaptic membrane (GO:0045211)	85 (2.17)	187 (0.99)	2.19	1.43E-08

^a^Num., number of proteins annotated.

^b^Per. percentiles of proteins annotated.

^c^E-ratio, enrichment ratio of bivalent genes.

Next, we calculated the distribution of bivalent genes in human ESC chromosomes, and found that ∼10–25% protein coding genes (23) in each chromosome are bivalent genes except the Y chromosome (Table 5). This distribution suggests that every chromosome may play a specific role related to pluripotency in ES cells. The Y chromosome is rich in junk (27) and has only 56 protein-coding genes (28), which may be the reason for just one bivalent gene found in Y chromosome. The same result can be achieved from the distribution of bivalent genes in mouse ESC chromosomes (Supplementary Table S3).

**Table 5.** Distribution for bivalent genes in human ESC chromosomes

Chromosome	Bivalent gene number	Protein-coding gene number	Percentiles (%)
1	366	2080	17.60
2	303	1333	22.73
3	221	1079	20.48
4	195	769	25.36
5	200	898	22.27
6	200	1054	18.98
7	182	983	18.51
8	165	702	23.50
9	193	829	23.28
10	185	774	23.90
11	228	1317	17.31
12	191	1070	17.85
13	83	332	25.00
14	123	866	14.20
15	121	619	19.55
16	142	886	16.03
17	223	1217	18.32
18	58	290	20.00
19	169	1496	11.30
20	128	562	22.78
21	47	247	19.03
22	103	511	20.16
X	86	836	10.29
Y	1	56	1.79

## Conclusion and Future Perspective

BGDB is the first attempt to establish a literature-based resource of bivalent genes by integrating genomic data, sequences, GO and other useful information. It is a valuable resource for better understanding the mechanism of gene expression regulation in pluripotent stem cells. Furthermore, the statistical analyses revealed functional diversity and enrichment of bivalent genes.

We will continuously maintain and update the database once new bivalent gene data are reported. Additionally, our next prospective goal is to collect and curate genes marked by H3K4me3 only, H3K27me3 only and neither H3K4me3 nor H3K27me3 in ES cells, respectively. This will make BGDB a more comprehensive resource for further study of ES cell epigenetics.

## Supplementary Data

Supplementary data are available at *Database* Online.

Supplementary Data
