# Supplementary material for: BGDB: a database of bivalent genes
Source: Database (Oxford). 2013 Jul 26;2013:bat057. doi: 10.1093/database/bat057 (PMC3724367; doi:10.1093/database/bat057)
Supplement: Supplementary Data [file supp_2013_bat057_index.html]

Supplementary Data 

# BGDB: a database of bivalent genes

## Supplementary Data

files

**Files in this Data Supplement:**

- Supplementary Data - doc file
