# Supplementary material for: BGDB: a database of bivalent genes
Source: Database (Oxford). 2013 Jul 26;2013:bat057. doi: 10.1093/database/bat057 (PMC3724367; doi:10.1093/database/bat057)
Supplement: Supplementary Data [file supp_bat057_Supplementary_data.doc]

**Supplementary Data:**

**BGDB: a database of bivalent genes**

Qingyan li^1^, Shuabin Lian^1^, Zhiming Dai^1^, Qian Xiang^1^, Xianhua Dai^1§^

^1^School of Information Science and Technology, Sun Yat-Sen University, Guangzhou 510006, China

^§^Corresponding author

*To whom correspondence should be addressed. Tel: +86-20-39943331;

Email: issdxh@mail.sysu.edu.cn.

**Supplementary Figure S1** – Four advance options in BGDB database. (A) Batch search allows users to input keyword list for querying; B) BLAST search. Users can input a gene or protein sequence to search identical or homologous sequences. (C) Browse allows users look through bivalent genes in a specific organism; (C) Download. Users can download whole database with MySQL or Excel format.


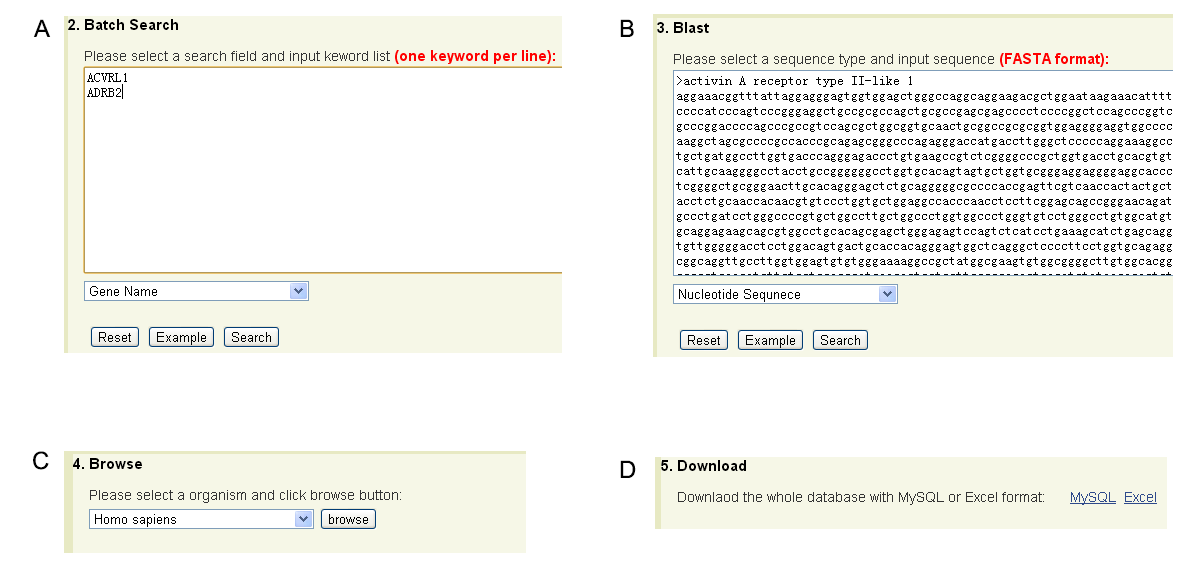


**Supplementary Table S1** – The top ten papers which contain most bivalent genes in our search result from PubMed.

| References | Gene no. |
| --- | --- |
| Pan *et al.* (1) | 3301 |
| Ku *et al.* (2) | 2978 |
| Mikkelsen *et al.* (3) | 2706 |
| Meissner *et al.* (4) | 2141 |
| Zhao *et al.* (5) | 1798 |
| Jia *et al.* (6) | 779 |
| Young *et al.* (7) | 419 |
| Hawkins *et al.* (8) | 355 |
| Vastenhouw*et al.* (9) | 236 |
| Bernstein *et al.* (10) | 135 |

**Supplementary Table S2** **-** The top five most enriched GO terms of biological processes, molecular functions and cellular components in mouse bivalent genes.

| Description of GO term | Bivalent Gene  n (%)^a,b^ | Genome  n (%) | E-ratio^c^ | P-value |
| --- | --- | --- | --- | --- |
| The top five most enriched biological processes |  |  |  |  |
| transport(GO:0006810) | 163 (5.46) | 137 (0.62) | 8.79 | 2.37E-72 |
| multicellular organismal development(GO:0007275) | 277 (9.28) | 310 (1.41) | 6.60 | 1.28E-103 |
| signal transduction(GO:0007165) | 213 (7.14) | 305 (1.38) | 5.16 | 4.73E-66 |
| anterior/posterior pattern specification(GO:0009952) | 69 (2.31) | 120 (0.54) | 4.25 | 9.71E-19 |
| nervous system development(GO:0007399) | 87 (2.92) | 156 (0.71) | 4.12 | 1.36E-22 |
| The top five most enriched molecular functions |  |  |  |  |
| hydrolase activity(GO:0016787) | 114 (3.82) | 136 (0.62) | 6.19 | 6.00E-41 |
| signal transducer activity(GO:0004871) | 136 (4.56) | 168 (0.76) | 5.98 | 1.86E-47 |
| sequence-specific DNA binding(GO:0043565) | 232 (7.77) | 568 (2.58) | 3.02 | 1.06E-40 |
| protein dimerization activity(GO:0046983) | 51 (1.71) | 133 (0.6) | 2.83 | 3.26E-09 |
| double-stranded DNA binding(GO:0003690) | 42 (1.41) | 114 (0.52) | 2.72 | 2.46E-07 |
| The top five most enriched cellular components |  |  |  |  |
| membrane(GO:0016020) | 661 (22.15) | 872 (3.96) | 5.60 | 6.74E-230 |
| neuron projection(GO:0043005) | 67 (2.25) | 150 (0.68) | 3.30 | 4.52E-14 |
| proteinaceous extracellular matrix(GO:0005578) | 73 (2.45) | 183 (0.83) | 2.95 | 3.11E-13 |
| cytoplasmic vesicle(GO:0031410) | 45 (1.51) | 103 (0.47) | 3.23 | 1.09E-09 |
| synapse(GO:0045202) | 65 (2.18) | 172 (0.78) | 2.79 | 4.18E-11 |

^a^Num., number of proteins annotated; ^b^Per. percentiles of proteins annotated; ^c^E-ratio, enrichment ratio of bivalent genes

**Supplementary** **Table S3** **–** Distribution for bivalent genes in mouse ESC chromosomes.

| Chromosome | Bivalent Gene no. | Protein coding gene no. | Percentiles (%) |
| --- | --- | --- | --- |
| 1 | 189 | 1240 | 15.24 |
| 2 | 256 | 1835 | 13.95 |
| 3 | 152 | 1059 | 14.35 |
| 4 | 180 | 1344 | 13.39 |
| 5 | 179 | 1302 | 13.75 |
| 6 | 170 | 1302 | 13.06 |
| 7 | 177 | 2053 | 8.62 |
| 8 | 161 | 1074 | 14.99 |
| 9 | 171 | 1264 | 13.53 |
| 10 | 135 | 1041 | 12.97 |
| 11 | 222 | 1649 | 13.46 |
| 12 | 126 | 838 | 15.04 |
| 13 | 127 | 872 | 14.56 |
| 14 | 105 | 1094 | 9.60 |
| 15 | 155 | 792 | 19.57 |
| 16 | 79 | 670 | 11.79 |
| 17 | 113 | 1095 | 10.32 |
| 18 | 107 | 493 | 21.70 |
| 19 | 91 | 720 | 12.64 |
| X | 89 | 942 | 9.45 |

**References**

1. Pan,G., Tian,S., Nie,J., Yang,C., Ruotti,V., Wei,H., Jonsdottir,G.A., Stewart,R. and Thomson,J.A. (2007) Whole-Genome Analysis of Histone H3 Lysine 4 and Lysine 27 Methylation in Human Embryonic Stem Cells. *Cell Stem Cell*, **1**, 299–312.

2. Ku,M., Koche,R.P., Rheinbay,E., Mendenhall,E.M., Endoh,M., Mikkelsen,T.S., Presser,A., Nusbaum,C., Xie,X., Chi,A.S., et al. (2008) Genomewide Analysis of PRC1 and PRC2 Occupancy Identifies Two Classes of Bivalent Domains. *Plos Genet*, **4**, e1000242.

3. Mikkelsen,T.S., Ku,M., Jaffe,D.B., Issac,B., Lieberman,E., Giannoukos,G., Alvarez,P., Brockman,W., Kim,T.-K., Koche,R.P., et al. (2007) Genome-wide maps of chromatin state in pluripotent and lineage-committed cells. *Nature*, **448**, 553–560.

4. Meissner,A., Mikkelsen,T.S., Gu,H., Wernig,M., Hanna,J., Sivachenko,A., Zhang,X., Bernstein,B.E., Nusbaum,C., Jaffe,D.B., et al. (2008) Genome-scale DNA methylation maps of pluripotent and differentiated cells. *Nature*, **454**, 766–770.

5. Zhao,X.D., Han,X., Chew,J.L., Liu,J., Chiu,K.P., Choo,A., Orlov,Y.L., Sung,W.-K., Shahab,A., Kuznetsov,V.A., et al. (2007) Whole-Genome Mapping of Histone H3 Lys4 and 27 Trimethylations Reveals Distinct Genomic Compartments in Human Embryonic Stem Cells. *Cell Stem Cell*, **1**, 286–298.

6. Jia,J., Zheng,X., Hu,G., Cui,K., Zhang,J., Zhang,A., Jiang,H., Lu,B., Yates III,J., Liu,C., et al. (2012) Regulation of Pluripotency and Self- Renewal of ESCs through Epigenetic- Threshold Modulation and mRNA Pruning. *Cell*, **151**, 576–589.

7. Young,M.D., Willson,T.A., Wakefield,M.J., Trounson,E., Hilton,D.J., Blewitt,M.E., Oshlack,A. and Majewski,I.J. (2011) ChIP-seq analysis reveals distinct H3K27me3 profiles that correlate with transcriptional activity. *Nucleic Acids Res.*, **39**, 7415–7427.

8. Hawkins,R.D., Hon,G.C., Lee,L.K., Ngo,Q., Lister,R., Pelizzola,M., Edsall,L.E., Kuan,S., Luu,Y., Klugman,S., et al. (2010) Distinct Epigenomic Landscapes of Pluripotent and Lineage-Committed Human Cells. *Cell Stem Cell*, **6**, 479–491.

9. Vastenhouw,N.L., Zhang,Y., Woods,I.G., Imam,F., Regev,A., Liu,X.S., Rinn,J. and Schier,A.F. (2010) Chromatin signature of embryonic pluripotency is established during genome activation. *Nature*, **464**, 922–926.

10. Bernstein,B.E., Mikkelsen,T.S., Xie,X., Kamal,M., Huebert,D.J., Cuff,J., Fry,B., Meissner,A., Wernig,M., Plath,K., et al. (2006) A Bivalent Chromatin Structure Marks Key Developmental Genes in Embryonic Stem Cells. *Cell*, **125**, 315–326.
